# Supplementary material for: Mechanisms governing the pioneering and redistribution capabilities of the non-classical pioneer PU.1
Source: Nat Commun. 2020 Jan 21;11:402. doi: 10.1038/s41467-019-13960-2 (PMC6972792; doi:10.1038/s41467-019-13960-2)
Supplement: Supplementary file 7 — Source data [file 41467_2019_13960_MOESM7_ESM.zip › Source_Data/FigureS2/FigureS2B_MotifScanOutput/KmeansCluster6/homerResults/motif4.similar.html]

motif4

## Information for motif4

C
T
G
A
A
T
G
C
C
G
A
T
C
A
G
T
A
G
T
C
C
G
A
T
A
T
C
G
G
A
T
C
G
C
A
T
G
C
A
T
G
A
C
T
G
A
C
T
  
Reverse Opposite:  

C
T
G
A
C
T
G
A
C
G
T
A
C
G
T
A
C
T
A
G
T
A
G
C
C
G
T
A
C
T
A
G
G
T
C
A
C
G
T
A
T
A
C
G
G
A
C
T
  

|  |  |
| --- | --- |
| p-value: | 1e-223 |
| log p-value: | -5.156e+02 |
| Information Content per bp: | 1.660 |
| Number of Target Sequences with motif | 662.0 |
| Percentage of Target Sequences with motif | 20.10% |
| Number of Background Sequences with motif | 1725.9 |
| Percentage of Background Sequences with motif | 4.01% |
| Average Position of motif in Targets | 98.2 +/- 42.3bp |
| Average Position of motif in Background | 98.3 +/- 76.6bp |
| Strand Bias (log2 ratio + to - strand density) | -0.0 |
| Multiplicity (# of sites on avg that occur together) | 1.06 |
| Motif File: | file (matrix) reverse opposite |

### Similar de novo motifs found

|  |  |  |  |  |  |  |  |
| --- | --- | --- | --- | --- | --- | --- | --- |
| Rank | Match Score | Redundant Motif | P-value | log P-value | % of Targets | % of Background | Motif file |
| 1 | 0.968 | A G T C C T G A A T G C C G A T A C G T A G T C C G A T T A C G A G T C G C A T G C A T G A C T G A C T G A C T | 1e-193 | -445.429958 | 11.05% | 1.09% | motif file (matrix) |
| 2 | 0.851 | A T G C A C G T A C G T A G T C A C G T A C T G A G T C | 1e-93 | -214.878174 | 40.57% | 23.69% | motif file (matrix) |
| 3 | 0.881 | A C G T A C G T A G T C A C G T A T C G A G T C C G A T C G A T A G C T | 1e-91 | -210.338291 | 13.18% | 3.98% | motif file (matrix) |
| 4 | 0.764 | C G T A T C A G C G T A C G T A A T C G C G T A C G T A A T C G C G T A C G T A A T C G G T C A C G T A | 1e-15 | -35.464259 | 2.37% | 0.75% | motif file (matrix) |
